# “Learning to Listen to Them and Ask the Right Questions.” Bennet Omalu, Scientific Objectivities, and the Witnessing of a Concussion Crisis

**DOI:** 10.3389/fspor.2021.672749

**Published:** 2021-07-21

**Authors:** Gregory Hollin

**Affiliations:** School of Sociology and Social Policy, University of Leeds, Leeds, United Kingdom

**Keywords:** autobiography, chronic traumatic encephalopathy, scientific objectivity, neuropathology, science and technology studies, standpoint theories

## Abstract

The death of American Football player Mike Webster has become foundational to narratives of sport's twenty-first century concussion crisis. Bennet Omalu, the neuropathologist who conducted Webster's autopsy and subsequently diagnosed Webster with Chronic Traumatic Encephalopathy (CTE), has, likewise, become a central figure in the concussion crisis. Indeed, it is frequently argued that there is something about Omalu in particular that made it possible for him to “witness” CTE when the disease entity had hitherto remained invisible to a great many medics and scientists. In this article, and drawing upon auto/biographies, I consider Omalu's self-described mode of scientific witnessing which purportedly allowed him to (re)discover CTE. I find Omalu's described objectivity to be shaped by three factors: First, the importance of “trained judgment” within which Omalu's scientific training is emphasized. Second, the infusion of religiosity within scientific practice. Third, a self-described position as an “outsider” to both football and American culture. Throughout this analysis, I pay attention not only to the ways in which Omalu's narratives depart from conventional depictions of scientific objectivity; I also note the similarities with particular bodies of social scientific work, most notably within a feminist “turn to care” in Science and Technology Studies (STS) and related standpoint epistemologies. Following these analyses, I argue that, first, Omalu's writing affords the dead a “response-ability” that is often absent within analyses of the concussion crisis and, second, that a focus upon diverse forms of objectivity, such as those described in Omalu's work, complements existing work into concussion science that has foregrounded scientific conflict of interest.

## Introduction: When Omalu Met Webster

There is now a substantial body of work that has examined the history of concussion in sport and that has sought to situate the contemporary concussion crisis more fully (Harrison, 2014; Casper, 2018a,b; Bachynski, 2019; Casper and O'Donnell, 2020). Scholars responsible for this work have convincingly argued that the ongoing concussion crisis has a long historical context: Harrison, for example, has shown that concussion has been discussed in relation to American Football for over a century (Harrison, 2014). Bachynski (2019) has likewise demonstrated that there has been a longstanding awareness of the significant safety issues associated with the sport, but argues that these fears have historically been counterbalanced by an individualization of risk on the one hand—the well-established mantra of “they know the risks”—and the stressing of individual and social benefit on the other. Casper, likewise, sees little evidence of a radical epistemic break in recent years and, despite some changes and consolidations across the twentieth century (Casper, 2018b), argues that since at least the 1870s there has been:

“…a continuity of clinical observations about HI [head injury] and a steady, incremental accumulation of knowledge refining our understanding of those observations from a remarkably wide sphere of scientific disciplines” (Casper, 2018a, p. 795)

In the context of this research, it is striking that perhaps the most widely established ‘origin story' of the concussion crisis does not even leave the twenty-first century: the posthumous diagnosis of Mike Webster with Chronic Traumatic Encephalopathy (CTE) by the neuropathologist Bennet Omalu.

The paper in which Webster's case was reported, entitled *Chronic Traumatic Encephalopathy in a National Football League Player* (Omalu et al., 2005) remains one of the most widely-cited papers in a growing field of research. Nonetheless, the influence of the Webster case study escapes such easy metrics. In 2019, a two-day conference entitled *The Neuropathological Diagnosis of Chronic Traumatic Encephalopathy (CTE): Next Steps*, hosted by the *National Institute of Neurological Disorders and Stroke* in Maryland brought together many of the world's leading CTE researchers. That the Webster case was the point of origin for this meeting is clear: Ann McKee, perhaps the most famed CTE researcher in the world, says during her presentation that:

“This is a case that broke it open, for the public, Omalu's case of Mike Webster… This was the beginning of the debate about CTE and certainly it triggered enormous interest in the disease, mine as well.” (McKee, 2019)

Speaking from the floor, a second neuropathologist named Rudolph Castellani says that, “I think we agree there is one case that got this discussion started. It was the Mike Webster case.”

The story of Omalu and Webster has also shaped societal understandings of CTE and concussion, in particular through the book *League of Denial* (Fainaru-Wada and Fainaru, 2013) and the film *Concussion* (Landesman, 2015), both of which center Omalu in the story of CTE. Ventresca has argued that these are the “key media texts” to have “crucially shaped” the “underlying tone of media coverage about CTE” (2019, p. 143) while Martin and McMillan describe *Concussion* as having “solidified star status for the fragile brain” (Martin and McMillan, 2020, p. 2). According to Sandel, *Concussion* may even have resulted in a drop in the popularity of the NFL (Sandel, 2020, p. 170).

In more fully understanding depictions of this encounter between Omalu and Webster, it is helpful to turn to a passage in *League of Denial* wherein Julian Bailes, a colleague of Bennet Omalu who also features in the film *Concussion*, draws a direct comparison between his early research on CTE and another public health crisis of the late-twentieth and early-twenty first century:

“…those early results would remind Bailes of another health crisis he had witnessed. “It was like when HIV started coming out; I was here in Chicago, and we didn't know what it was,” he said. “There were these young men, 22, 23 years old, showing up with Kaposi's sarcoma and other weird things that you shouldn't get when you're 23. It was the HIV suppressing their immune system.”

Looking back, Bailes believed that he and [co-author] Barry Jordan had stumbled onto “the first whiff” of another new disease.” (Fainaru-Wada and Fainaru, 2013, p. 69).

Thinking across HIV/AIDS and CTE in this manner, Webster in many ways figures as “patient zero,” taking the role occupied by Gaëtan Dugas in the HIV/AIDS epidemic (McKay, 2017). Omalu, meanwhile, is positioned as a pioneering medic akin to William Darrow (see Bell et al., 2019, chapter 2 for an approach that thinks across HIV/AIDS and CTE in relation to media coverage).

One of my central arguments in this paper is that, in the case of CTE discourse, the figuring of the doctor and the patient during this encounter are very different to one another. While Webster is the first individual to be (posthumously) diagnosed with CTE, he and his suffering is positioned as completely non-exceptional; there were others before him and others after him who, but for happenstance, could easily have received the first diagnosis. The place of Mike Webster in this story could be exchanged for Junior Seau, or Chris Benoit, or Jeff Astle and the story of the concussion crisis would, in all likelihood, have unfolded in a very similar manner. This is not the case for Bennet Omalu. While he too has been followed by a list of illustrious peers—Ann McKee, Willie Stewart, Chris Nowinski—it is frequently argued that it was far more than fortuitous timing that led to his initial witnessing of CTE: there was something very particular about him that facilitated these observations. In other words, while the temporal ordering of Junior Seau and Mike Webster is held to be epistemologically irrelevant to understanding CTE, it is a similarly widely articulated argument that Ann McKee *could only* have become interested in CTE *after* Omalu.

In the following sections, I seek to interrogate the underpinnings of this rendering of Bennet Omalu as somehow exceptional by examining depictions of Omalu's subject- and object-ivity, as described in auto/biography. After first detailing existing scholarship on scientific objectivity and the role of auto/biography therein, I go on to argue that within narratives concerning Omalu three themes intertwine: First, Omalu describes his objectivity in terms akin to the notion of “trained judgment” within which his scientific training is emphasized. Second, Omalu describes his religiosity as infusing his scientific practice. Finally, third, Omalu self-describes a distinct position as an “alien” or “outsider” to both football and American culture.

Throughout the above analysis I pay attention not only to the ways in which Omalu's narratives depart from conventional depictions of scientific objectivity but also note the similarities with particular bodies of social scientific work, most notably that within a feminist “turn to care” in Science and Technology Studies (STS) (Puig de la Bellacasa, 2011, 2017) and related standpoint epistemologies (e.g., Harding, 1993). In the conclusion, I make two points. First, I argue that Omalu's writing affords the dead a “response-ability” (Haraway, 2016, p. 78, Haraway, 2008, chapter. 3) that is often absent within analyses of the concussion crisis. Second, I argue that a focus upon diverse forms of objectivity, such as those described in Omalu's work, complements existing work into concussion science that has foregrounded scientific conflict of interest. In particular, I suggest that a focus on objectivity may draw attention to the possibility that scientists situated within diverse epistemic cultures (Knorr Cetina, 1999) may witness the concussion crisis in disparate ways, not simply because of explicit biases but because of commitments built into practices themselves.

## Diverse Objectivities

In order to grasp the significance of Omalu's described mode of scientific objectivity, it is important to first engage with wider debates about the historical construction of ‘scientific objectivity' and the concomitant emergence of particular forms of scientific subjectivity. In the landmark text *Objectivity*, Daston and Galison trace the emergence of scientific objectivity to the mid-nineteenth century, detailing in particular a mode of objectivity that they name *mechanical objectivity*. For those scientists enacting mechanical objectivity there is an:

“…insistent drive to repress the willful intervention of the artist-author, and to put in its stead a set of procedures that would, as it were, move nature to the page through a strict protocol, if not automatically.” (Daston and Galison, 2010, p. 121).

Thus, mechanical objectivity necessitates an active attempt to suppress or remove the hand of the scientist from scientific outputs. Daston and Galison call this form of scientific witnessing “blind sight” (see-ing without a see-er) and there are clear resonances here with other historical analyses of the sciences that have, for example, understood the modern scientist as a “modest witness” (e.g., Shapin and Schaffer, 1985).

Daston and Galison argue that, from the late nineteenth century, there has been a splintering of objectivity so that a variety of distinct forms are now evident. For example, a number of scholars developed a variant of objectivity known as “structural objectivity.” For these “ascetics among ascetics” (Daston and Galison, 2010, p. 259), objectivity is grounded “in structures rather than images” (Daston and Galison, 2010, p. 254)—in particular the structures of mathematics and/or logic that survive theory-change and are most evidently observed today in in analytic philosophy and mathematical physics. Quite differently, the notion of “trained judgment” also emerged as a form of objectivity around the turn of the twentieth century. The “trained judgment” approach emphasized the importance of training when it comes to producing and understanding scientific work. Scholars advancing the notion of trained judgment recognized the importance of gut feelings and hunches in scientific discovery and argued for the importance of training in order to procure insight (Daston and Galison, 2010, p. 311). Finally, toward the turn of the twenty-first century, Daston and Galison argue that scientific fields such as nanotechnology shift away from a form of objectivity premised upon on observing nature, or intervening in nature, and, rather, focus on making: the merging of the “camera and the tweezer” (Daston and Galison, 2010, p. 397). In this emerging mode of objectivity, Nature itself is reconstituted: it ceases to pre-exist scientific activity and is, instead, forged in, and through scientific activity.

Science and Technology Studies scholars have gone on to elucidate various other forms of objectivity. Cambrosio and colleagues, for example, have coined the term “regulatory objectivity” (Cambrosio et al., 2006, 2009) to denote a form of objectivity within which regulation—for example “schemes, guidelines, and models of action” (Cambrosio et al., 2009, p. 655)—are crucial to the undertaking of scientific research. Others have turned away from academia entirely. Murphy, for example, has understood the medical self-help practices of feminists during the 1970s—vaginal self-examination, for example (Murphy, 2012, p. 73)—in relation to the history of objectivity, describing feminists as “lay researchers” practicing a form of “immodest witnessing.” Murphy persuasively argues that these self-help practices underpin alternative, normative forms of objectivity practiced and advocated within quarters of the social sciences (Murphy, 2012, p. 98), most notably feminist standpoint theories such as Harding's “strong objectivity” (Harding, 1993) that have been articulated as alternatives to approaches arising from the natural sciences (See also: Haraway, 1988, 1997).

There are a number of important points that follow from these historical analyses. First, there is no tight binding between “objectivity” and “science”: Science was being practiced before the elucidation of mechanical objectivity in the nineteenth century and, likewise, lay researchers within feminist self-help groups were practicing forms of objectivity outside of the institutions of science. Second, to speak of a singular, intransigent objectivity is a dubious proposition given science's diverse epistemic cultures (Knorr Cetina, 1999). Diverse forms of objectivity—mechanical, structural, regulatory, and so forth—have come into being and, if twenty-first century sciences like nanotechnology and synthetic biology (Roosth, 2017, pp. 27–28) are indicative, will continue to do so. Third:

“[o]bjectivity and subjectivity are as inseparable as concave and convex; one defines the other. The emergence of scientific objectivity in the mid-nineteenth century goes hand in glove with the emergence of scientific subjectivity.” (Daston and Galison, 2010, p. 197)

Thus, different forms of scientific objectivity stand in relation to different forms of scientific subjectivity. The scientist of mechanical objectivity was formed in relation to the “post-Kantian” self; the structural objectivist was shaped by findings within “science itself, particularly the then-young sciences of sensory physiology and experimental psychology” (Daston and Galison, 2010, p. 258); while trained judgment was influenced by the emerging sciences of the unconscious.

## Autobiographies and Technologies of the Self

Autobiographies have been understood to play a small but significant part in the constitution of scientific object- and subject-ivities. For Daston and Galison, the importance of autobiographical texts arises because, and following Foucault (e.g., Foucault, 1984, 2005; see Kelly, 2013 for an overview), they understand the cultivation of particular forms of scientific subjectivity as progressing, in part, through a variety of technologies of the self. Specifically:

“The kinds of practices that we will be concerned with include training the senses in scientific observation, keeping lab notebooks, drawing specimens, habitually monitoring one's own beliefs and hypotheses, quieting the will, and channeling attention. Like Foucault, we assume that these practices do not merely express a self; they forge and constitute it.” (Daston and Galison, 2010, p. 199)

As might be expected, these technologies and techniques of the self vary alongside the forms of objectivity and subjectivity being cultivated.

Scientific autobiography is a technology of the self that came to play an interesting role in performances of object- and subject-ivity during the establishment of mechanical objectivity during the nineteenth century. The blind sight required for the enactment of mechanical objectivity necessitated that the modest witness remove any traces of themselves from scientific outputs and, within that context, the auto/biography provided an avenue for scientists to find their voice:

“Certainly the literary conventions of scientific publications as they gradually developed over the course of the eighteenth and nineteenth centuries do seem to have erased more and more of the author's personality and circumstances. At the same time, other genres [such as autobiography] emerged and proliferated that reconnected lives and works in science.” (Daston and Galison, 2010, p. 217)

This is not to say that scientists have approached their autobiographies with a free hand, for there “exists a widespread fear [in science] that the release of the subjective voice will distort or even smother its objective counterpart” (Shortland, 1988, p. 171) and, thus, there remained an incentive to “*marginalise* the creative involvement of individual scientists” within autobiography (Shortland and Yeo, 1996, p. 9, italics in original).

Analysis of scientific auto/biography, then, should not be mistaken with analysis of scientific practice, for these are documents intended to both cultivate and pontificate when it comes to forms of objectivity and subjectivity. Understood in these terms, however, auto/biographies can be incredibly insightful, not as windows onto the world but “precisely *as* historically specific stereotypes and moral lessons” about how the scientists in question think that science should be conducted (Daston and Galison, 2010, p. 232 italics in original). It is in this vein, that I now turn to works by and about Bennet Omalu in order to better understand the self-described conditions of possibility that facilitated his initial witnessing of CTE and the moral lessons contained within that framing.

## Omalu's Autobiographies

Abir-Am argues that:

“Scientists usually get involved in autobiographical endeavours because they either make or miss major discoveries. By their own account, such efforts are coupled with a desire to “set the record straight,” presumably because some of their colleagues have already set it crooked.” (Abir-Am, 1991, p. 326)

Bennet Omalu fits this model perfectly: His (re)discovery of CTE through the case of Mike Webster is evidently a major discovery that, as detailed above, has solidified its status as the point of origin for a whole academic discipline. Similarly, Omalu understands himself as having been “marginalized, minimalized, [and] ostracized” (Omalu, 2017, chapter 18) within the contemporary field, most notably in the work of his one-time colleague Chris Nowinski and Ann McKee at Boston University.

Omalu has written a full-length autobiography, *Truth Doesn't Have a Side* (2017), and an additional text, *Play Hard, Die Young* (2008), that contains significant autobiographical elements. In addition to these self-penned works, there are a number of (much more widely consumed) texts that contain lengthy discussions with and about Omalu. Most prominent amongst these texts are the books *League of Denial* (Fainaru-Wada and Fainaru, 2013) and *Concussion* (Laskas, 2015), the latter of which tells Omalu's story over 260-odd pages, was based upon an article in *GQ Magazine*, features long passages voiced by Omalu, and appeared as a feature film starring Will Smith (Landesman, 2015).

As mentioned above, these texts have been extremely influential to the public understanding of concussion and CTE. Here, though, I ask not after the influence of these texts but, instead, consider the forms of objectivity and subjectivity enacted within them. If auto/biographies can indeed be considered “moral exemplars” in their performance of object- and subject-ivity (Daston and Galison, 2010, p. 232) and if, as outlined above, there is understood to be something very particular about Omalu's mode of scientific practice, then understanding that practice may contribute to our understanding of the contours of the contemporary concussion crisis. In the following analysis, I highlight three aspects of these texts in particular: first, Omalu's training as an expert scientist; second, the entanglement of Omalu's science and his religion; third, his self-described status an outsider: an outsider status that Omalu describes as being both racialized and affecting his subsequent position within the nascent scientific field. I argue that, in the coming together of these themes, a distinctive mode of scientific objectivity is being elucidated.

## Analysis

### Trained Judgment

The first theme, and one largely to be expected, in depictions of Omalu's mode of witnessing is the assertion that his scientific training and expertise was, in a conventional sense, essential to his identification of CTE in Mike Webster. Omalu's career trajectory—from the medical school at the University of Nigeria, to the University of Washington in the United States, to hospitals in New York and Pittsburgh, to further degrees from the University of Pittsburgh and Carnegie Mellon (Fainaru-Wada and Fainaru, 2013, p. 154)—is seen as illustrative of an exceptionally intelligent man who “seemed to collect degrees and certificates with the ease of a man picking out produce at the supermarket” (Fainaru-Wada and Fainaru, 2013, p. 149). Likewise, Omalu's manual skills during dissection, and ability to recognize similarities and resonances between his patients, is foregrounded during *League of Denial* (Fainaru-Wada and Fainaru, 2013, pp. 9–10). In short, and as Omalu says:

“…my medical background and training placed me at the right place at the right time and equipped me to recognize these cases and these diseases when I saw them. What the mind does not know, the eyes do not see…” (Omalu, 2008, p. 2)

This is a conventional story of scientific witnessing, perhaps most closely aligning with the form of objectivity described by Daston and Galison as “trained judgment” wherein the “trained expert (doctor, physicist, astronomer) grounds his or her knowledge in guided experience, not special access to reality” (2010, p. 359). Indeed, Omalu's decision to examine Webster's brain in the absence of visible pathology, a decision seemingly made on the spur of the moment (Fainaru-Wada and Fainaru, 2013, p. 10), similarly adheres to this mode of scientific work that, as discussed earlier, leaves significant room for hunches and gut instinct (Daston and Galison, 2010, p. 311).

If Omalu's diagnosis of CTE in Mike Webster were described as being entirely predicated upon his trained judgement, then there would not be much of a story to tell. Certainly, it would be hard for Omalu and his interlocuters to make the argument that only he could have diagnosed Webster, for similar tales of exceptional scientific aptitude could surely be—indeed have been (e.g., Leavy, 2012)—told about scientists such as McKee, Nowinski, and Bailes. What makes Omalu particular, however, is the infusion of both religion and cultural distance into his science.

### Religion and Response-Ability

In his own work, Omalu makes repeated reference to religion and scripture. While a number of historical scientific autobiographies retain traces of religiosity (Outram, 1996, p. 93), and a great number of influential scientists have emphasized their religion, surveys of autobiography in the contemporary biological and neurological sciences report little in the way of religious musing (Abir-Am, 1991; Söderqvist, 2002). Similarly, within the monumental collection *The History of Neuroscience in Autobiography*, an endeavor currently spanning many thousands of pages and seven volumes published between 1996 and 2011 (e.g., Squire, 2011), there is scant reference to the supernatural, religion, or God; and discussion is largely negative when the topic does arise.

Omalu, by contrast, saturates narratives of both his personal and professional life with references to God and religion. Omalu uses religious turns of phrase both to understand his own life circumstances—a period of struggle and suffering is described as “my Calvary” (Omalu, 2017, p. 27)—and the conversion of others to his perspective likewise—“the doubting Thomases repented and become liberated into the light of the scientific truth” (Omalu, 2008, p. 90). Chapters titles in his autobiographies are overtly religious—e.g., “In the name of Christ, stop!” (Omalu, 2017, chap. 16)—and scripture is frequently quoted, e.g., “‘.you will learn the truth, and the truth will set you free.' John 8:32” (Omalu, 2008, p. 131). For present purposes, however, what is more significant than these personal understandings and rhetorical devices are the instances where Omalu describes his religion and spirituality—“a fusion of Roman Catholicism and Igbo tribal mysticism”—as becoming “entwined with his medical practice” (Fainaru-Wada and Fainaru, 2013, p. 149).

This entanglement is perhaps most evident in Omalu's descriptions of autopsy. Omalu describes his role in preparing a body for autopsy as being akin to “a servant who prepared his master for the great beyond by the cleansing of the autopsy, as though this was a vital step in the transition from earth to heaven” (Omalu, 2017, p. 99). Given this framing of autopsy as religious ceremony, it is perhaps unsurprising for Omalu to note that the autopsy “became a spiritual experience for me” (Omalu, 2017, p. 94).

Omalu describes an important part of the process of autopsy as talking to the deceased person. Thus, the corpse is not, as might be expected, treated by the pathologist as an “I” that has “become an object” (Kristeva, 1982, p. 13), but, rather, continues to be treated as a subject. Omalu says, for example, that:

“The players who have suffered and probably died from this disease [CTE] want to be heard even from the land of the dead. Their spirits may be yearning to communicate with us to share their own stories.” (Omalu, 2008, p. 136)

Here, then, we get a sense that when Omalu says “what the mind does not know, the eyes do not see” he is not simply talking about the identification of neuropathological disease through biomedical expertise. Because the spirits of the diseased were “yearning to communicate” with him, the trained judgment of Omalu went beyond conventional modes of scientific witnessing, for Omalu was also “learning to listen to them [the spirits, the diseased patients] and ask the right questions” (Omalu, 2017, p. 95).

There is no sense that the listening and two-way communication described here by Omalu should be understood as anything but literal. In a scene toward the end of the film *Concussion*, Omalu addresses the NFL Players Association Concussion Meeting and states that “I wish I had never met Mike Webster. But that was before I knew him. He has given me a great gift, a dangerous gift, a gift of knowing” (Landesman, 2015). As Omalu speaks, he looks up and sees the ghost of Mike Webster sitting quietly amongst the audience. While it is tempting to dismiss this piece of fiction simply as Hollywood melodrama, Omalu states quite clearly, in non-fiction, that the dead do speak to him and that he sees ghosts in his coroner's office (Fainaru-Wada and Fainaru, 2013, p. 149).

The authenticity of these conversations and interactions—as well as the stakes involved in the plea with which this scene in *Concussion* concludes, the plea for family members to “Forgive them. Forgive yourselves. Be at peace.”—is perhaps most evident in Omalu's descriptions of Chris Benoit. Benoit was a professional wrestler who, in 2007, killed his wife and son before killing himself, and who was posthumously diagnosed by Omalu as suffering from CTE (Omalu et al., 2010. For an overview of the case and Benoit's life see: Shoemaker, 2014, pp. 349–367; For analysis of Benoit's legacy within contemporary professional wrestling, see: Desilets, 2019). Omalu kept the brains of various patients in “the coat closest in my condominium” and Benoit was no exception in this regard (Omalu, 2017, pp. 196–197). While working on Benoit's case, and with his brain in the closest, the deceased Benoit is described by Omalu as vandalizing his car and tampering with his household appliances in an attempt to ensure Omalu tells his story (Omalu, 2017, pp. 149, 209; Omalu, 2008, pp. 136–137). While Omalu's wife is, understandably, perturbed by these supernatural events, Omalu says simply that:

“I smiled and asked her not to worry about it. Not every ghost is evil. We have good ghosts. With her in the kitchen, I spoke out loud, addressing thin air, and said, “Please, I promise you. I will do what I can do to help.” I do not think he meant any harm.” (Omalu, 2008, p. 137)

Throughout the process of autopsy and neuropathological analysis, then, Omalu describes himself as listening to, and interacting with deceased persons—a conversation enacted through spirits in “thin air” and their embodied, earthly, remains. Importantly, within both Omalu's own narratives, these acts are central to his discovery of CTE for it is only through them that Omalu is able to “…listen to them and to ask the right questions” (Omalu, 2017, p. 95). Knowing, in other words, is predicated upon conversation with the dead.

Omalu's descriptions of his practice are, by all accounts, quite alien to both the scientific auto/biographical tradition and to his American colleagues. Unsurprisingly, therefore, the descriptions also mark a clear point of departure from the forms of scientific “modest witnessing” described in the introduction. Omalu's descriptions, though, do find clear resonances with work within the social sciences. In particular, there are semantic, and quite possibly substantive, links here with the “turn to care” within contemporary STS and the posthumanities more generally (See in particular the work of Puig de la Bellacasa, 2011, 2017).

Within the body of thought that has concerned the “turn to care,” Barbara McClintock, a Nobel Prize winning cytogeneticist who worked extensively with maize, has long been understood as enacting an alternative mode of scientific research more in keeping with social scientific and feminist praxis (e.g., Keller, 1984). McClintock's science is in many ways captured by her belief that:

“I'm beginning to suspect that a large part of the research has been done with the ulterior motive of imposing an answer on it [the object of enquiry]… if only we were content to let the material speak!” (McClintock in, Stengers, 1997, p. 126)

Rather than objectifying the materials of scientific activity, it is argued that McClintock cared deeply about her subject matter, lived and worked in close proximity to the material (a factor deemed to be of particular significance by proponents of this approach, e.g., Greenhough and Roe, 2011), and was open to hearing the material speak. Following Despret, Haraway (2008, pp. 88–89) has deemed this approach one in which there is an openness to “response-ability” wherein the object of enquiry is afforded the opportunity to “speak back” and “impose their own “requirements” on the scientist” (Candea, 2013, p. 109). Despret's own work in this regard concerns animal science and she argues explicitly against “those who made the animal into a soulless machine” and is instead in favor of recognizing agency in unexpected places (Despret, 2016, p. 7). Despret's book is called *What Would Animals Say if We Asked the Right Questions?*, a question with very obvious resonances with Omalu's own plea “…to listen to them and ask the right questions” (Omalu, 2017, p. 95).

This is not the only time where, within Omalu's auto/biographies, we can find resonances with social scientific research agendas. In particular, I argue below, Omalu also appears to approach his topic with a distinct ethnographic gaze and engages in conceptual work with distinct affinities to Sandra Harding's formulation of “strong objectivity” (Harding, 1993).

### Participant Observation, Conformational Intelligence, and Strong Objectivity

A re-occurring feature in Omalu's auto/biographies is a profound sense of alienation—from American Football as a sport but also from American culture writ large. Omalu writes, for example, of seeing a game of American Football on television during his childhood in Nigeria:

“…it was unlike any game I had ever witnessed. The players dressed up like extraterrestrials…[t]hey looked like broad-chested, big-headed, tiny-legged visitors from Mars. The game didn't make a lot of sense to me.” (Omalu, 2017, p. 34)

This confusion does not recede upon Omalu's arrival in the United States. In Pittsburgh, for example, Omalu describes attending a party where all the other guests are engrossed in a Steelers game:

“.this feeling that I had just witnessed some sort of odd religious sect or cult, like an anthropologist who stumbles upon a ritual ceremony in a clearing in the middle of a rain forest under a starry sky.” (Omalu, 2017, p. 142)

As this quote makes clear, it was not simply the on-field practices that made Omalu feel “like an alien” (Omalu, 2017, p. 215), but American culture more generally.

Omalu describes his positioning as a black, African man as central to this sense of alienation from American culture. Shortly after arriving in the country, Omalu describes a dawning realization that he is being treated differently to “white folks” around him: he is followed by suspicious staff while shopping and is the only student to be stopped by the police while walking home at night (Laskas, 2015, p. 51). Omalu says that as “…a child in Nigeria, we were not taught about racism. As a man from Nigeria, until I began to experience these behavioral patterns, I was not mentally aware of the concept of racism” (Laskas, 2015, p. 51; See also: Omalu, 2017, pp. 70–74).

Omalu describes talking through these personal experiences of racism with his pastor and reading about the history of slavery in the United States—both in general and in specific relation to Igbo peoples (Laskas, 2015, pp. 54–55, 68)—and articulates a growing belief that the “place I thought God had blessed more than any nation on earth… was no different from any other place on earth” (Omalu, 2017, p. 72). These experiences, writes Omalu, fed directly into his science and he describes a hardening of his resolve to challenge conventional thinking:

“… every time I smelled racism. I even became angrier and more determined to be myself and stand for what I believe in, which in my mind was the truth. I did not need anyone to legitimize me. I recognized I was an outsider. Racism even made me better at that.” (Laskas, 2015, p. 193)

Omalu's invocation of himself as an outsider can here usefully be read against his alternative rendering of himself as a stereotyped anthropologist observing an “odd religious sect.” The anthropologist, as an ethnographer, is not strictly an outsider but, rather, a *participant* observer. This is the anthropologist's paradox; “the contradictory idea of being both within and without the worlds we study” (Pandian, 2019, p. 26). This provides context for a line in *League of Denial* wherein it is said that Omalu “looked at the game through the lens of a Martian, if that Martian happened to practice neuropathology” (Fainaru-Wada and Fainaru, 2013, p. 151). Here, the biographers dwell upon Omalu's alienness and his status as outsider while simultaneously making us aware that, if he were not at home in the neuropathology laboratory, then CTE would never have been discovered in Mike Webster (See also: Laskas, 2015, p. 124).

The importance of this alienation is centralized in Omalu's own depictions of his work on CTE and his decision to look for the disease in the brain of Mike Webster:

“Was a professional football career similar to a boxer's when it came to head injury? To the untrained eye – which Bennet definitely was when it came to both American Football and boxing – it seemed similar.

What the mind doesn't know, the eye can't see. But what the mind knows too well, the eye can miss altogether.” (Laskas, 2015, p. 124)

As Omalu says himself:

“I discovered CTE in an American Football player only because I was an outsider whose thinking about that sport – and all contact sports – did not conform to the accepted ideas within the culture of this country. Because of this, my eyes looked for what others had not.” (Omalu, 2017, p. 230)

Intriguingly, Omalu refers to this outsider status as a form of objectivity, repeatedly stating that “I may have been more objective in approaching and treating the NFL cases I prosecuted because of my lack of sentimental involvement with football” (Omalu, 2008, p. 4) and that it “took an outsider like myself, who did not conform to America's cast of the mind about football, to objectively link Mike Webster's ailments to football and identify CTE” (Omalu, 2017, p. 48).

This question of “America's cast of the mind” leads directly to Omalu's conceptual contribution to this matter. Omalu argues that:

“The leading neurological and neuropathological researchers in the best academic and research centres in the country [the United States] did not discover something that should have been very obvious for at least as long as football players wore plastic helmets and turned what should have been protection into a weapon.” (Omalu, 2017, p. 219)

Put bluntly, Omalu believes the reasons for the inability of American scientists to discover CTE is that “No one who truly loves football would want to recognize anything negative about the sport” (Omalu, 2008, p. 12).

Omalu settles on the name “conformational intelligence” to describe this phenomenon. Two passages in particular provide a good summary of the term “conformational intelligence.” First:

“I define conformational intelligence as a phenomenon whereby the way you think and perceive the world, including your sense of right and wrong and good and evil, are controlled, constrained, and constricted by the expectations, cultures, traditions, norms, and mores of the society around you without you even knowing it or being aware of it. As a result, when objective, factual evidence is presented to you that runs counter to the conformational cast of your mind, you deny and reject that evidence, even though it is true and your preconceived ideas are false.

Some people have told me that if I had grown up in this country and become consumed by the conformational intelligence surrounding football, there was no way I could have performed an autopsy on Mike Webster. I would have been in so much awe of him that I would not have touched his body.” (Omalu, 2017, pp. 47–48)

Second:

“When you are a member of a group, the group influences your mentality, your presuppositions, and therefore your way of thinking and processing information, without you even being aware of it. You reach the same conclusion as the rest of the group, even when that conclusion is not supported by science. This occurs over and over with physicians connected to the sports industry. They become so intoxicated by the status, fame, and exclusivity of their connection to their sport that they become zombies without even realizing it. As someone who stepped in and observed this from the outside, I have though this to be an interesting phenomenon.” (Omalu, 2017, p. 214)

These two passages certainly have their differences. In the first passage, conformational intelligence in the context of American Football appears to apply to Americans *tout court*: Omalu insists that “if I had grown up in this country” he may well have been “consumed” by conformational intelligence, plausibly suggesting that the American “cast of the mind” develops from a young age. In the second quotation, the accusation of conformational intelligence is far more closely targeted at those American scientists “connected to the sports industry.” What the passages do have in common, however, is a continual focus on outsider status as key to insight; it is not Omalu's intelligence *per se* that is key to his success but, rather, the particular form of relation that he has with American Football and with America.

Within this context, it is worth noting that the agenda-setting conference mentioned in the introduction, *The Neuropathological Diagnosis of Chronic Traumatic Encephalopathy: Next Steps*, may have re-affirmed Omalu's work on the Webster case as foundational, but the scientist himself was notably absence. Indeed, by 2019, Omalu's absence was a familiar one, for there “was no place for Bennet Omalu in this brave new world” (Fainaru-Wada and Fainaru, 2013, p. 290) of consensus meetings and conferences with the NFL. While scientists involved in these meetings offer a variety of academic reasons for Omalu's exclusion (Fainaru-Wada and Fainaru, 2013, pp. 292–293; Hobson, 2020), Omalu himself understands conformational intelligence and his status as a racialized outsider as key to a continued inability to access the corridors of power. In response to an article in *The Washington Post* that included quotes from several of the world's pre-eminent CTE scientists and was entitled “from scientist to salesman: how Bennet Omalu, doctor of ‘Concussion' fame, built a career on distorted science” (Hobson, 2020), for example, Omalu asks why “…The Washington Post will choose to denigrate, diminish, ridicule and dismiss the work of a black immigrant physician like myself I do not know,” before going on to claim that the author of the piece “…wants you to see only his prejudiced perspective that I am a black villain, an uncivilized thug who should not have had the intelligence to be a doctor or discover a disease his preferred American doctors could not discover” (Omalu, 2020, paras. 8, 14). Similar passages are evident throughout Omalu's autobiographies. Omalu says that he was told that he did not have “the believeability factor” (Fainaru-Wada and Fainaru, 2013, p. 252; Laskas, 2015, p. 173) and, positively quoting a colleague, suggests that “They [former colleagues] want to replace your black face with that of a blonde-headed white woman [Ann McKee] with whom they are more comfortable” (Omalu, 2017, p. 212). Omalu goes on to suggest that there is a general desire to “tune out that crazy Nigerian and keep watching and playing like usual” (Omalu, 2017, p. 230).

It was noted in the preceding sub-section that Omalu's attempt to afford both corpses and spirits a response-ability contains notable affinities with work within feminist science studies that has advocated for a “turn to care.” This body of thought is significantly influenced by feminist standpoint theories (e.g., Puig de la Bellacasa, 2011, p. 95, 2017, p. 14) and it is thus intriguing to note further similarities between Omalu articulations of his own subjectivity and that body of thought.

In attempting to understand the preponderance of androcentric and sexist assumptions in science (see, e.g., Martin, 1991; Haraway, 1992 for classic analyses), Harding offers the following rationale:

“…the methods and norms in the disciplines are too weak to permit researchers *systematically* to identify and eliminate from the result of research those social values, interests, and agendas that are shared by the entire scientific community or virtually all of it. Objectivity has not been “operationalized” in such a way that scientific method can detect sexist and androcentric assumptions that are “the dominant beliefs of an age” – that is, that are collectively (versus only individually) held.” (Harding, 1993, p. 52 italics in original)

Thus, for Harding, while the laboratory sciences are able to identify inter-laboratory *difference* they are less able to identify a systemic source of bias (the believed inferiority of women, for example) when it is *shared* by an entire research community (Harding, 2015). As a corrective to this systemic bias, Harding calls for a “strong objectivity” which departs from the more usual forms of objectivity noted earlier (Daston and Galison, 2010) in that emancipatory ideals are built into scientific research by “socially situating knowledge projects in the scientifically and epistemologically most favorable historical locations” (Harding, 1993, p. 53). Building upon Marxist, but also postcolonial (Willey, 2016), traditions, the “most favorable historical locations” here are occupied by those such as the proletariat, women, or people of color who have the least interest in maintaining current, unjust conditions (Harding, 1991, p. 59).

Unsurprisingly, there are clear differences between standpoint theorists and Omalu. For example, the experience of living under oppression has been key for the epistemic privilege associated with the immodest witnessing of feminist activists (Murphy, 2006, pp. 63–64) while Omalu describes a clear detachment from the workings of football^1^. Nonetheless, Omalu's formation of “conformational intelligence,” the assertion that the “expectations, cultures, traditions, norms, and mores of the society” impact upon scientific research without any conscious awareness on the part of the scientists undoubtedly aligns with Harding's belief that shared “social values, interests, and agendas” need to be combated with forms of strong objectivity.

## Discussion

In this paper, I have sought to make the following arguments. First, within both scientific and popular discourse, the encounter between Bennet Omalu and Mike Webster, wherein the former diagnosed the latter with CTE, has become consolidated as the foundational origin story for the contemporary concussion crisis in sport. Second, while Mike Webster occupies the position of “patient zero” in this narrative, it is Omalu who is understood to be crucial to discovery: another football player could have been the first to be diagnosed, but Omalu and only Omalu could have made this first diagnosis. Third, and building upon work in the history of science (Daston and Galison, 2010), I examined this claim of exceptionalism on the part of Omalu by exploring the particular modes of subject- and object-ivity foregrounded in Omalu's auto/biographies. I argued that these texts elucidated a mode of scientific witnessing that foregrounded, first, Omalu's scientific training; second, his religiosity and a willingness to engage in conversation with the bodies and spirits of his patients and; third, his racialized “outsider status” which meant that he avoided the “conformational intelligence” of American scientists who were blinded to the harms of contact sport. In detailing these arguments, I have noted various affinities with existing bodies of social scientific research, most notably that from ethnography, the “turn to care” in science studies, and feminist standpoint theory. In understanding Omalu's mode of objectivity, therefore, I have suggested that his position bares as many similarities with Harding's “strong objectivity” (Harding, 1993) as it does those approaches more conventionally found in the sciences.

It is worth reiterating the nature of the claims I am making here. First, I have not been involved in a search for origins (Foucault, 1977, p. 140) but rather have sought to analyse an origin *story*. I am less invested in whether Omalu really was the only person who could have diagnosed CTE in Mike Webster than with the fact that this assertion has become a widely articulated part of contemporary narratives. I have sought to understand Omalu's auto/biographies as offering “moral lessons” about the nature of what the author takes to be good science (Daston and Galison, 2010, p. 232) rather than windows onto scientific practice.

Second, and following others in the social studies of science (e.g., Rees, 2016), my analysis has involved recognizing Omalu as author, methodologist, and theoretician in his own right (Fitzgerald, 2017, p. 182). My analysis has thus not only involved identifying but also taking seriously his claims and contributions, culminating in the counter-positioning of his own work to a widespread “conformational intelligence.” However, I do, and for two reasons, want to avoid falling into the trap of taking Omalu's claims uncritically. First, within Omalu's work there is undoubtedly a sense that ideology is what happens to other people. Omalu is highly critical of those who take research money from the NFL and, at times, suggests researchers' complicity in a significant CTE cover-up. Omalu is dismissive of those who gain financially from a pitch side diagnostic tool known as “Immediate Post-Concussion Assessment and Cognitive Testing” or “ImPACT” (Omalu, 2017, p. 194). He did not seem to worry unduly, however, about the possibility that either fame or the founding of a company called *Taumark*, which aimed toward a CTE diagnostic—and whose research partners were singled out for criticism by the Food and Drug Administration (Belson, 2015)—would negatively affect his work (Omalu, 2017, p. 236). Indeed, the auto/biographies examined here are not simply moral lessons in science, but commodities that have the potential to benefit Omalu economically and socially^2^. Quite differently, the gender politics of the texts under consideration here often leave a lot to be desired. The descriptions of Omalu's wife Prema largely adhere to the biographical trope of women as “…tolerant spouses, delighted with opportunity to subordinate their lives… to the demands of their scientist husbands' overwhelmingly important careers…” (Abir-Am, 1991, p. 342), while the re-occurring refusal to name “the woman” (Omalu, 2017, p. 214) who now occupies a prominent place in the field—Ann McKee—is likewise problematic, reaffirming through its singularity McKee's exceptional place in a male dominated profession.

With these provisos in mind, I would like to conclude with the following two points. First, in a recent special edition of the journal *a/b: Auto/Biography Studies* devoted to her work, Donna Haraway wrote “It matters what thought think thoughts, what stories tell stories, what knowledges know knowledges…” (Haraway, 2019, p. 570). For Huff, writing in the same special edition, the promise of specifically Harawayian modes of story- and knowledge-telling is that if we:

“…look at knowledge as situated, as part of a natureculture web, we decentre the human lives and expand the very concept of what knowledge is and what constitutes a life to include beings beyond the human and to consider death as well as life” (Huff, 2019, p. 377)

Accordingly, scholars influenced by Haraway's work are producing stories that actively seek to include and recognize the voices and stories of the dead and non-human (for particularly vivid examples, see, e.g., Kohn, 2013; Van Dooren, 2014).

It is within this context that I turn to a recent thematic analysis of three films (*Concussion, League of Denial, and Head Games*) dealing with the head injury (Bell et al., 2019, chap. 7). One of the key themes that the authors identify in these films is “humanizing the NFL player.” The authors note that, despite these films' attempts at humanization, a:

“…glaring omission in the discussion [within these films] was the players… the faces of CTE were most often left for secondary sources to tell their stories and guide discussion because the primary source was dead.” (Bell et al., 2019, p. 110)

Within this quote, there is a straightforward alignment with death and silence and it is taken as self-evident that the dead cannot speak (even if those who knew them are able to recount old stories from life). Furthermore, the suggestion that the dead do not “tell their stories” in these films elides the fact that Mike Webster is *not* silent in *Concussion* after his death: as noted above, he is in the audience at the NFL Players Association Concussion Meeting, and gives tacit approval to Omalu's speech. Conversations with Mike Webster, Chris Benoit, and others occur in all of the texts analyzed in this essay (including the book of *League of Denial*) and Omalu explicitly states that they are guiding his scientific practice. This guidance from the dead is possible because, as Omalu phrases it, he has learned to listen and ask the right questions. In Haraway's terminology, Omalu affords the dead a “response-ability” (Haraway, 2016, p. 78, 2008, chap. 3) that is denied them in many stories and apparently deemed impossible in the aforementioned analysis.

My argument here is not that, despite many similarities with standpoint epistemologies and care ethics, Omalu's mode of storytelling is identical to that proposed by Haraway. Instead, and following Huff's call for a decentring of the human when studying auto/biographical texts, I am suggesting that recognizing the dead's response-ability within Omalu's texts, and the ways in which that response-ability is associated with Omalu's particular mode of subjectivity, is important to understanding the descriptions of his objectivity and science. Further, and when telling our own stories about concussion and CTE, I suggest that Omalu's writing offers an invitation to think about our own modes of storytelling and who is permitted to speak within those stories.

Second, The science of CTE is frequently articulated as being incredibly fractious. Within popular science (e.g., Sandel, 2020, chap. 7), social science (e.g., Bachynski and Goldberg, 2014; Partridge, 2014; Baugh et al., 2020; Malcolm, 2020, chap. 4), and popular media (e.g., Doward, 2020) it is overt conflicts of interest that are most frequently highlighted and discussed. Perhaps the most common term of reference here is Big Tobacco's attempts to cover up the harmful effects of smoking: this is a comparison made both in the texts analyzed here (Fainaru-Wada and Fainaru, 2013, p. 6; Laskas, 2015, p. 164; Omalu, 2008, p. 43) and in other, diverse, pieces (e.g., Bachynski, 2019, p. 155; Endedijk and van Steenbergen, 2020). That historian of science Naomi Oreskes—author of *Merchants of Doubt*, one of the leading texts on various industries' attempts to cover up public health crises—put her name to a piece making this comparison seems to show that it is not being made lightly (see: Casper et al., 2019). Indeed, as academic research has shifted in recent decades to more fully embed industry partners—a shift elucidated through concepts such as “Mode 2” knowledge production (Nowotny et al., 2003) and the “triple helix” of the academy, state, and industry (Etzkowitz and Leydesdorff, 2000)—affinities with cigarette science have been reported across an array of contemporary scientific fields, including those investigating climate change (Oreskes and Conway, 2010), pharmaceuticals (Dumit, 2012, pp. 106–109), and fracking (Wylie, 2018, p. 69).

Biographies and autobiographies of Bennet Omalu do little to dissuade of the relevance of these analyses. Indeed, it can't get much clearer than Omalu's chapter title “The NFL = big tobacco” (Omalu, 2017, chap. 15). It remains, of course, crucial that scholarship continue to analyse what is sometimes termed “the sports-industrial-complex” and that where conflicts of interest are found these relationships are investigated and interrogated. Nonetheless, “conformational intelligence” suggests something beyond overt acts of bias. Rather than the lurking suggestion that there is a binary between “good” and “bad,” or “pure” and impure,” science, Omalu's concept points us toward social norms that inflect all research within a field and that, *qua* Harding, are much harder for members of a research community to “systematically identify and eliminate” (Harding, 1993, p. 52).

Some work within the social sciences has begun to move in just such a direction, moving beyond straightforward analyses of conflict of interest. Grano, for example, has recently argued that brain banking, a significant mode of scientific witnessing during the concussion crisis, is likely to “reify neoliberal conceptions of personalized risk management and empowered choice” (Grano, 2020, p. 341). Grano further argues that the social values and conceptions of risk embedded within brain banking are unlikely to do justice to classed and raced aspects of risk-based decision making that are key to understanding the concussion crisis. Others (e.g., Brayton et al., 2019; Henne and Ventresca, 2019; Martin and McMillan, 2020) have likewise identified reductionist and/or neoliberal logics underpinning reporting into the concussion crisis; a conclusion that chimes with existing research suggesting that neuroscientific findings frequently perpetuate rather than challenge existing understandings of society (O'Connor et al., 2012; O'Connor and Joffe, 2013). These conclusions regarding the underpinning logics of concussion science and journalism are complementary to, and yet notably distinct from, those that consider overt conflicts of interest.

As with the aforementioned scholars' analyses, my focus here on Omalu's self-described mode of subject- and object-ivity, eschews the binary of “biased” and “unbiased” science and instead focuses on diverse epistemic cultures (Knorr Cetina, 1999) and the particular ethico-epistemic commitments of these sciences.

This assertion that there is “no view from nowhere” need not be read as post-structural nihilism. It is certainly consistent with some branches of standpoint theory to suggest that some locales offer epistemically privileged vantage points. A question for social scientists investigating the concussion crisis, as with those studying other areas where industry significantly shapes research, is how to embed these privileged vantage points, and emancipatory values, within sites of knowledge creation and dissemination. In other empirical areas, STS scholars have approached this task through a range of means, including taking faculty positions within the life-sciences (e.g., Roy, 2018) and the founding of novel organizations outside of existing institutional arrangements (e.g., Wylie, 2018). Omalu's writing, which as described above has both methodological and theoretical resonances with existing social scientific though, hints at the possibility of finding willing partners in these future endeavors.

## Data Availability Statement

The original contributions presented in the study are included in the article/supplementary material, further inquiries can be directed to the corresponding author/s.

## Ethics Statement

Written informed consent was not obtained from the individual(s) for the publication of any potentially identifiable images or data included in this article.

## Author Contributions

The author confirms being the sole contributor of this work and has approved it for publication.

## Conflict of Interest

The author declares that the research was conducted in the absence of any commercial or financial relationships that could be construed as a potential conflict of interest.

## Footnotes

^1^In this sense at least, it Chris Nowinski, who frames his own journey into CTE research in relation to his own brain trauma (Nowinski, 2007) and Ann McKee, who's fandom has long been noted within both academic (Grano, 2020, p. 344) and popular texts (Almond, 2014, pp. 54–57) who are closest to the immodest witnesses demanded of some standpoint theories. For many, including both Omalu and those cited in the previous sentence, the closest of these scholars to sports' governing bodies represents not a position of epistemic privilege—as Murphy argues is was the case for feminist medical self-help groups (Murphy, 2012)—but a position with possible conflicts of interest.

^2^Thank you to an anonymous reviewer for pointing out this final point.
